# Electrochemical properties of a novel EDLC derived from plasticized biopolymer based electrolytes with valuable energy density close to NiMH batteries

**DOI:** 10.1038/s41598-023-48417-6

**Published:** 2023-11-30

**Authors:** Shujahadeen B. Aziz, Mohamad A. Brza, Rebar T. Abdulwahid, Jamal Hassan, Hawzhin B. Tahir, Sameerah I. Al-Saeedi, Ranjdar M. Abdullah, Jihad M. Hadi

**Affiliations:** 1 Research and Development Center, University of Sulaimani, Qlyasan Street, Sulaimani, Kurdistan Regional Government 46001 Iraq; 2 Department of Physics, College of Science, Charmo University, Chamchamal, Sulaymaniyah 46023 Iraq; 3 Medical Laboratory Analysis Department, College of Health Sciences, Cihan University Sulaimaniya, Sulaymaniyah, Kurdistan Region 46001 Iraq; 4 Department of Physics, College of Education, University of Sulaimani, Old Campus, Sulaymaniyah, Kurdistan Region 46001 Iraq; 5 Department of Physics, Khalifa University, P.O. Box 127788, Abu Dhabi, United Arab Emirates; 6 Department of Chemistry, College of Science, Princess Nourah bint Abdulrahman University, P.O. Box 84428, 11671 Riyadh, Saudi Arabia; 7 Nursing Department, College of Nursing, University of Human Development, Sulaymaniyah, Kurdistan Regional Government Iraq

**Keywords:** Energy science and technology, Materials science

## Abstract

This study introduces a novel system of solid electrolytes for electrical double-layer capacitors (EDLCs) utilizing biopolymer electrolytes with high energy density comparable to NiMH batteries. To prepare the electrolytes, a proton-conducting plasticized chitosan: poly(2-oxazoline) (POZ) with good film-forming properties was fabricated using a solution casting technique, and ammonium trifluoromethanesulfonate (NH_4_CF_3_SO_3_) salt was employed as a proton provider. Various glycerol concentrations were incorporated into the chitosan:POZ: NH_4_CF_3_SO_3_ system to enhance the ionic conductivity and fully transparent films were obtained. The impedance technique was utilized to determine the conductivity and measure the diffusion coefficient, mobility, and number density of ions. The electrochemical measurements, including linear sweep voltammetry (LSV) and cyclic voltammetry (CV), validated the high performance of the system. The EDLC was examined using galvanostatic charge-discharge (GCD) equipment, and the results revealed an energy density of 43 Wh/kg, specific capacitance of 300 F/g, and power density of 1800 W/kg over 500 cycles. These findings suggest that it is plausible to develop EDLCs that resemble batteries, making them a more desirable energy storage option for the industry.

## Introduction

Polymer electrolytes (PEs) play an essential role in the functionality of electrochemical devices such as electric double-layer capacitors (EDLCs) and proton batteries. They enable efficient ion transport, which improves stability, performance, and safety, thereby establishing their potential as energy storage materials. The development of solid polymer electrolytes (SPEs) began in 1979 using lithium batteries^1^. Liquid electrolytes (LIs) are eminent owing to their good performance in energy storage devices^2,3^. However, LI evaporates easily and harms the equipment owing to corrosive and leaking issues^4^. SPEs are a good replacement for LIs owing to their long shelf life, easy fabrication, and safety^5^. Lithium batteries provide very good performance and conductivity; however, they degrade naturally and cause environmental pollution^6^. Thus, researchers started to use H^+^ ions, including NH_4_^+^ ions, instead of Li-ion providers^2,7^. Incorporating polymer blending during the preparation of PEs is a valuable method that increases the availability of sites for ion exchange and hopping^8^ and enhances the mechanical strength and thermal stability of the resulting PEs^9^.

In this study, chitosan (CS) polymer was chosen for its affordability and natural abundance. CS serves as a host for ion conduction due to its structural composition and can form complexations with inorganic salts due to its amino and hydroxyl groups^10,11^. Polymers with electron donor groups can dissolve low- lattice energy inorganic salts through weak coordination bonds to prepare PEs. The poly(oxazoline) (POZ) monomer having O atoms and N atoms is responsible for making complexation in POZ-based PEs^12,13^. Due to the electron donor group in the POZ backbone, it is a suitable polymer for the preparation of PEs and polymer blending.

Conductivity is increased by loading salts into polymer blends. For example, the ammonium triflate (NH_4_CF_3_SO_3_) salt addition into poly(ethyl methacrylate) (PEMA) based PEs improved the conductivity from 8.6 × 10^–11^ S/cm to 1.02 × 10^–5^ S/cm^14^. Earlier studies^15–18^, revealed that CS based electrolytes are successful for EDLC applications. The significance of CS polymer is that it is an environmentally friendly, non-toxic, natural polymer whose sources are biomaterials. It is important to clarify that the NH_4_CF_3_SO_3_ salt has acted as a proton donor, increasing the number of protons in the electrolyte. As a result, this improvement has increased the efficiency of charge transfer and conductivity. The ammonium salts are proper proton donors to the PEs and less cost-effective compared to lithium salts, which are toxic and produce deterioration at the electrode/electrolyte interface^19,20^. Rodi et al. measured an ionic conductivity of 7.20 × 10^–8^ S cm^–1^ by adding 20 wt.% of LiCF_3_SO_3_ in PEMA based PEs at room temperature (RT)^21^. Based on literature^22–24^ ammonium salts that have NH_4_^+^ cations are crucial for PE preparation because under the AC field one of the weakly attached H^+^ of the NH_4_^+^ cation can easily detach and move and thus produce high ionic conductivity. Hopefully, the size of H^+^ is less than the Li^+^ cation and thus, H^+^ will be the focus of many research groups in the near future for electrochemical device applications.

**Figure 1:**
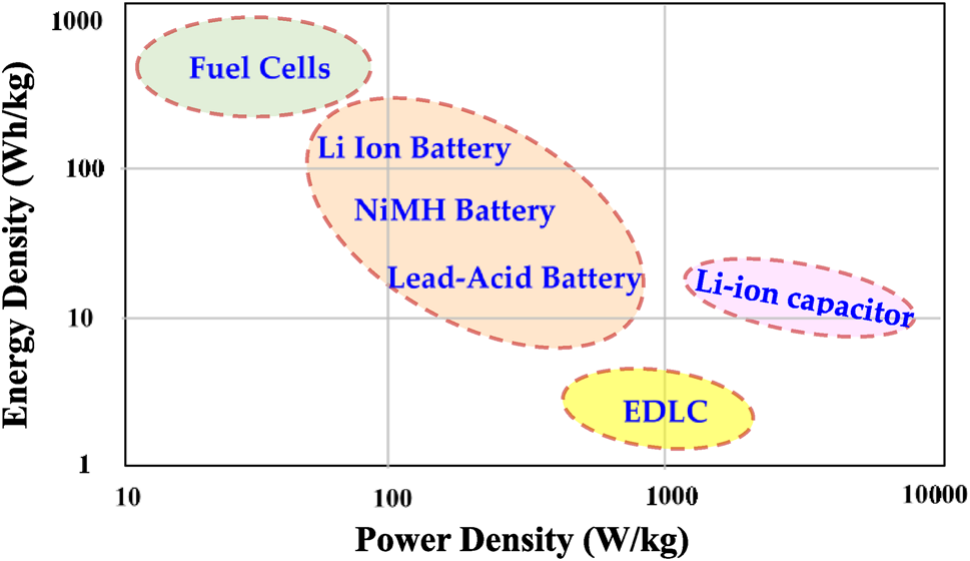
The Ragone plot for various electrochemical devices.

The present work prepares the blend polymer with a load of NH_4_CF_3_SO_3_ salt as a proton donor for the PEs and the glycerol. Glycerol weakens the electrostatic force between anions and cations, causing more salts to dissociate into free ions^25^. The PE is used in the fabrication of the EDLC device where the process of energy storage takes place by ions accumulation at the blocking electrodes and electrolyte interfaces^26^. EDLC has high cyclability, good power density, the same carbonaceous electrode, and a reasonable lifetime^27^. Several reports in the literature used SPEs in the synthesis of EDLC^28–31^. According to the prospective use of PEs in EDLC assemblies, they have a high specific capacitance comparable to liquid and gel-based electrolytes. To ultimately replace battery technology with a non-toxic alternative, the main goal of incorporating PEs into EDLC devices is to attain an energy density comparable to batteries. Nonetheless, the fundamental obstacle to EDLC commercialization continues to be their poorer energy density when compared to batteries. As a result, research teams and companies working on energy storage are now concentrating on creating EDLCs with high energy densities greater than 30 Wh/kg. Discussions about the future of EDLC technology will probably be sparked by the study's findings. The electrolyte used in the EDLC assembly also displayed a relatively high energy density, higher than lead-acid batteries and comparable to NiMH batteries, as illustrated in Fig. 1.

This investigation presents an EDLC device that exhibits excellent performance throughout 500 cycles, with high energy and power densities. The effect of glycerol on conductivity at room temperature is demonstrated through electrical measurements utilizing EIS. The electrolyte ion transport and dielectric properties are also discussed in detail.

## Preparation and characterization of SPE

### Preparation of the electrolytes

**Figure 2:**
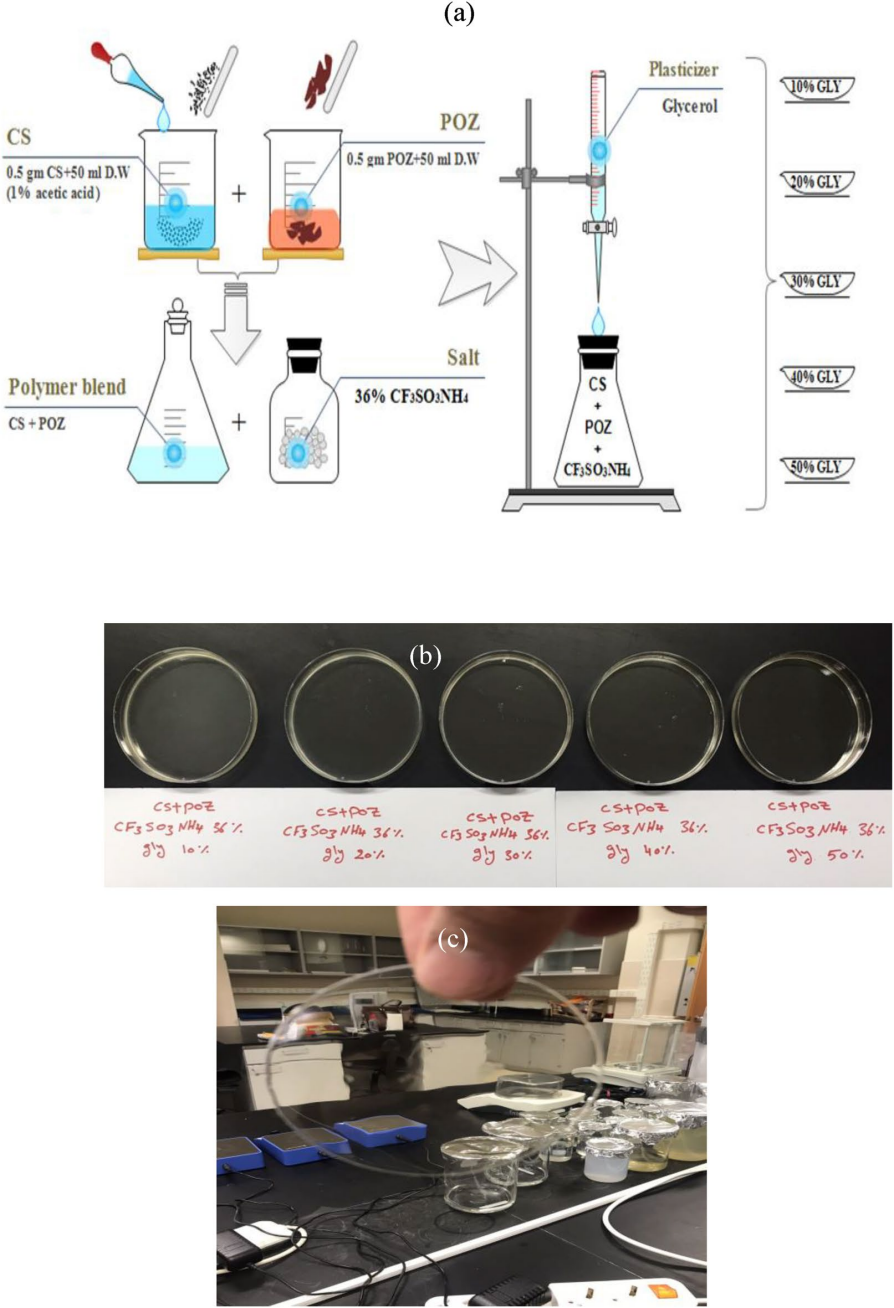
(**a**) Schematic diagram of the plasticized film preparation, (**b**) realistic images of the plasticized samples after solvent evaporation, and (**c**) Image of a fully transparent highest plasticized sample. The thickness of SPE films is ~280 µm.

The combination of CS and POZ was generated through the solution casting method. This process involves dissolving 80% weight of CS in 100 mL of 1% acetic acid, and 20% weight of poly (2-ethyl-2-oxazoline) (POZ) in 20 mL of distilled water. These two solutions were mixed and stirred with a magnetic stirrer until a uniform blend was achieved. Following that, 50% weight of NH_4_CF_3_SO_3_ was added to the CS:POZ solution and the mixture was stirred until a consistent solution of CS:POZ: NH_4_CF_3_SO_3_ PEs was formed. The solution was then blended with glycerol in the amounts specified in Table 1, and the samples were designated accordingly. Finally, the solution was poured into Petri dishes and dried at room temperature. The films were further dried using desiccators. The schematic diagram of the plasticized film preparation, realistic images of the plasticized samples after solvent evaporation, and an image of the fully transparent highest plasticized sample are shown in Fig. 2a–c.

**Table 1:** The composition of the electrolyte samples.

Sample designation	CS (wt. %)	POZ (wt. %)	NH_4_CF_3_SO (wt. %)	Glycerol (wt. %)
CSPCFSN1	50	50	36	10
CSPCFSN2	50	50	36	20
CSPCFSN3	50	50	36	30
CSPCFSN4	50	50	36	40
CSPCFSN5	50	50	36	50

### Electrolyte investigations

#### EIS method

Impedance spectra at RT are achieved with HIOKI 3532-50 LCR Hi-tester in the frequency between 50 and 2,000,000 Hz and a couple of stainless steel (SS) electrodes used to sandwich the SPE during measurement.

#### LSV and CV studies

The linear sweep voltammetry (LSV) analysis is essential to the proper use of CS:POZ:NH_4_CF_3_SO_3_:Gly electrolytes in EDLCs. These techniques provide valuable information on the stability, potential window, and ionic conductivity of the electrolyte, enabling the calculation of the EDLC’s operating potential and overall performance.

Using CS:POZ:NH_4_CF_3_SO_3_:Gly electrolytes in EDLCs requires thoroughly examining their LSV properties. LSV is a technique that provides information on the stability and potential window of the electrolyte. This information is crucial in determining the working potential of the EDLC. The LSV analysis is performed using a potentiostat, which measures the current response of the electrolyte to a changing potential. The sample used in LSV analysis consists of the highest conductive CS:POZ:NH_4_CF_3_SO_3_:Gly electrolyte and two stainless steel disks. The cell is enclosed in a Teflon case to ensure that the measurement is not influenced by external factors.

### Fabrication and analysis of the EDLC

**Figure 3:**
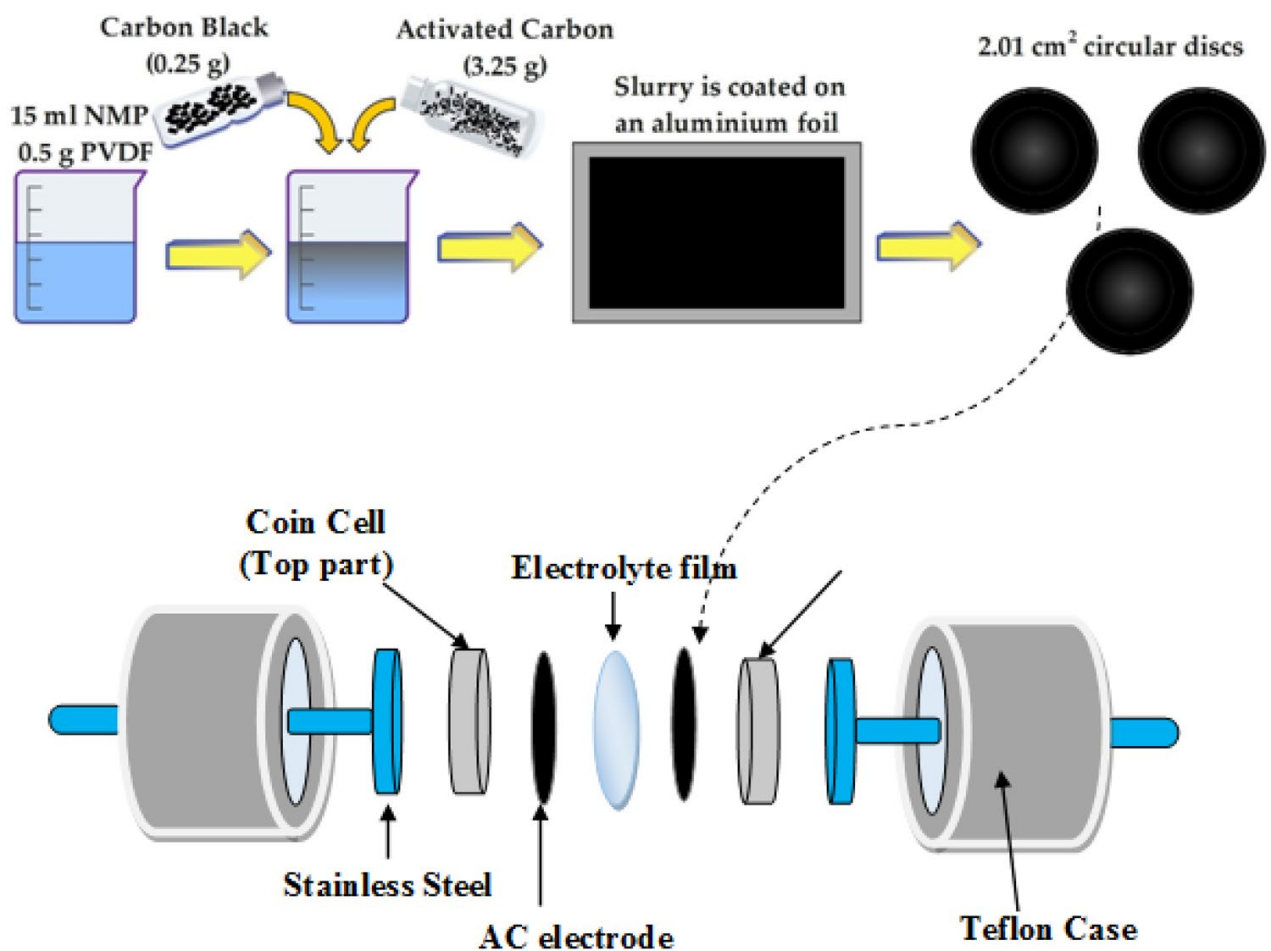
AC electrodes preparation process and illustration of assembled EDLC.

Figure 3 depicts the process of preparing AC electrodes and assembling an EDLC. The planetary ball mill blended carbon black (CB) and AC, creating a fine powder. PVdF served as the binder, and NMP was the solvent. The CB-AC mixture was added to the NMP and PVdF solution, and slow stirring for 5 hours with a magnetic stirrer was performed to prevent excess bubbles. This resulted in a black and thick slurry. After cleaning with acetone, the slurry was coated on a flat piece of aluminum foil laid out on a glass surface using a doctor blade. The electrodes were dried in an oven at 60 °C for a few hours, cooled to room temperature, and placed in a desiccator for further drying. A 2.01 cm diameter circular shape was cut as the electrode. The EDLC device was formed by filling the space between the AC electrodes in the CR2032 coin cell with a maximum conductivity electrolyte. The EDLC’s properties were verified using CV tests at slow (10 mV/s) and fast (100 mV/s) scan rates. The specific capacitance (*C*_*s*_) was calculated using Eq. (1) from the CV data:

1 $$ C_{s} = \int_{{V_{i} }}^{{V_{f} }} {\frac{I\left( V \right)dV}{{2ma\left( {V_{f} - V_{i} } \right)}}} . $$

Here, through Origin 9.0 software, the area of the CV plot area (*I(V)dV*) was measured. *m*,* a*, *V*_*f*_ and *V*_*i*_ are the mass of the electrode, scan rate, final voltage, 1.0 V, and the initial voltage, which 0 V, respectively.

## Results and discussion

### Electric impedance spectroscopy (EIS) study

**Figure 4:**
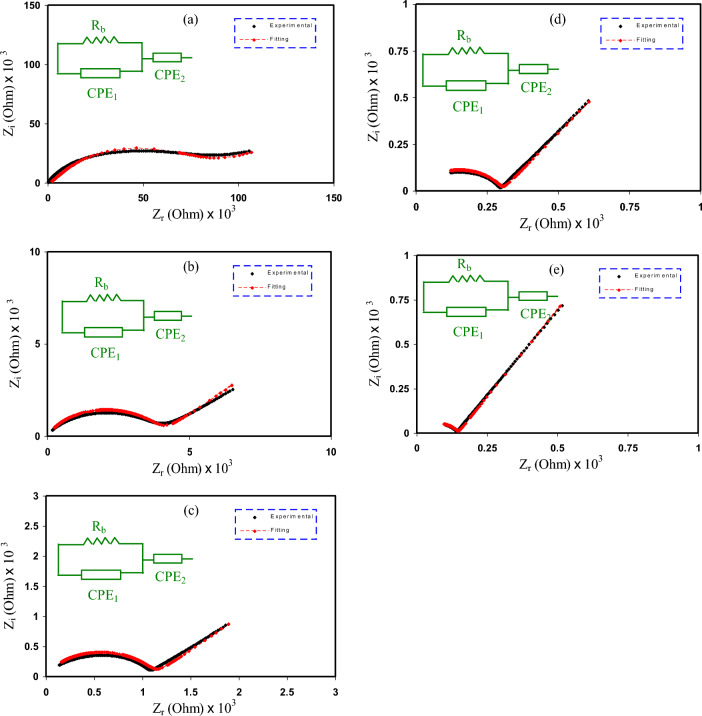
EIS data for the samples considered in this work (**a**) CSPCFSN1, (**b**) CSPCFSN2, (**c**) CSPCFSN3, (**d**) CSPCFSN4, and (**e**) CSPCFSN5.

Figure 4a–e shows the Cole-Cole plot for the samples at room temperature. Results show a distinct low-frequency spike and a semicircular high-frequency curve. The bulk resistance (R_b_) is precisely determined by the point at which the curve’s tail and the real axis connect. Ionic conduction in the PE sample’s main body causes the semicircle. However, the spike seen in the figure indicates ion polarization caused by the existence of a space charge layer near the electrodes, which in turn causes an increase in the low-frequency range^32^. From Fig. 4a–e, it is evident that the semicircle decreases as the glycerol content in the sample increases to 50 wt. %. This is an indication that the addition of glycerol improves the ionic conductivity. The decrease in the semicircle indicates a decrease in the resistance of the films. The increase in ionic conductivity that occurred when glycerol was added to the polymer matrix can be ascribed to its function as a plasticizer. By increasing the chain mobility and free volume of the polymer, glycerol generates additional channels for ion movement. This process enhances the ease of ion transport within the material, leading to a reduction in bulk resistance. The sample with the highest glycerol concentration (CSPCFSN5) has the lowest R_b_ value at room temperature, implying that it has the best overall conductivity among the samples.

To analyze the impedance spectra, the electrical equivalent circuit (EEC) model was employed^33^. The EEC is a valuable technique for interpreting impedance data, particularly when a spike and a semicircle appear in the plot. In this work, the EEC was illustrated (see the inset of Fig. 4) as a parallel connection between R_b_ and a constant phase element (CPE_1_) in series with another constant phase element (CPE_2_), as detailed in reference^34^. The CPE in the EEC serves as a capacitor substitute and compensates for any inhomogeneities in the system. This is because the CPE can be adjusted to represent the heterogeneous properties of the sample, thereby improving the accuracy of the impedance analysis. The impedance of the CPE, Z_CPE_, can be expressed by an equation, providing a quantifiable representation of the impedance behavior^34–36^:

2 $$ Z_{CPE} = \frac{1}{{C\omega^{p} }}\left[ {\cos \left( {\frac{\pi p}{2}} \right) - i\sin \left( {\frac{\pi p}{2}} \right)} \right], $$

where p, ω, and C are the Cole-Cole plot deviation from the axis, angular frequency, and capacitance of CPE, respectively. The real (Z_r_) and imaginary (Z_i_) parts of impedance associated to the EEC are given by:

3 $$ Z_{r} = \frac{{R_{b}^{2} C_{1} \omega^{p1} \cos \left( {\frac{{\pi p_{1} }}{2}} \right) + R_{b} }}{{2R_{b} C_{1} \omega^{p1} \cos \left( {\frac{{\pi p_{1} }}{2}} \right) + R_{b}^{2} C_{1}^{2} \omega^{2p1} + 1}} + \frac{{\cos \left( {\frac{{\pi p_{2} }}{2}} \right)}}{{C_{2} \omega^{p2} }}, $$

4 $$ Z_{i} = \frac{{R_{b}^{2} C_{1} \omega^{p1} \sin \left( {\frac{{\pi p_{1} }}{2}} \right)}}{{2R_{b} C_{1} \omega^{{^{p1} }} \cos \left( {\frac{{\pi p_{1} }}{2}} \right) + R_{b}^{2} C_{1}^{2} \omega^{2P1} + 1}} + \frac{{\sin \left( {\frac{{\pi p_{2} }}{2}} \right)}}{{C_{2} \omega^{p2} }}. $$

In these equations, p_1_, p_2_, C_1_, and C_2_ represent the semicircle deviation from the Z_i_ axis, deviation of the tail from the real axis, high-frequency capacitance, and low-frequency capacitance, respectively. The parameters of the EEC are presented in Table 2.

**Table 2:** The fitting parameters in EEC.

Sample	P_1_ (rad)	p_2_ (rad)	CPE_1_ (F)×10^–8^	CPE_2_ (F)×10^–5^
CSPCFSN1	0.79	0.31	0.556	0.323
CSPCFSN2	0.78	0.52	0.909	1.33
CSPCFSN3	0.79	0.53	1.25	4.00
CSPCFSN4	0.81	0.63	1.28	4.55
CSPCFSN5	0.81	0.7	1.35	4.76

To determine the Direct Current (DC) conductivity (σ_dc_) of the PEs, the area (A), thickness (t), and bulk resistance (R_b_) are used in the following equation.

5 $$ \sigma_{dc} = \left( {\frac{1}{{R_{b} }}} \right) \times \left( \frac{t}{A} \right). $$

Table 3 displays the σ_dc_ values of the PEs considered in this work. Results indicate an increase in conductivity upon increasing the concentration of glycerol. This is because glycerol provides free ions to the host polymer, which increases the flow of electricity and hence an increase in conductivity values. It has been reported^37,38^ that a conductivity of about 10^–4^ S/cm is appropriate for implementation in electrochemical energy storage systems, such as capacitors and batteries. In our work, the conductivity of the sample with 50 wt. % glycerol (CSPCFSN5) has reached 1.34×10^–4^ S/cm, indicating that it can be used for energy storage devices.

**Table 3:** The conductivity of the PEs obtained using Eq. (5).

Sample	Conductivity (S/cm)
CSPCFSN1	2.25×10^–7^
CSPCFSN2	3.93×10^–6^
CSPCFSN3	1.40×10^–5^
CSPCFSN4	5.14×10^–5^
CSPCFSN5	1.34×10^–4^

To further examine the samples, various additional parameters, including mobility (μ), self-diffusion coefficient (D), and number density (n) of ions, are obtained using the following equations^39^:

6 $$ D \, = \left( {\frac{{(K_{2} \varepsilon_{o} \varepsilon_{r} A)^{2} }}{{\tau_{2} }}} \right), $$

where *τ*_*2*_ is the reciprocal of ω, which is similar to the least value of *Z*_*i*_.

7 $$ \mu \, = \left( {\frac{eD}{{K_{b} T}}} \right) $$

where *T* is the temperature in Kelvin, and *k*_*b*_ is the Boltzmann constant.

8 $$ n \, = \left( {\frac{{\sigma_{dc} K_{b} T\tau_{2} }}{{(eK_{2} \varepsilon_{o} \varepsilon_{r} A)^{2} }}} \right). $$

The obtained values of these parameters are presented in Table 4. As seen, their values increase with the concentration of glycerol in the samples.

**Table 4:** The parameters of the PEs at RT, using the equations given in the text.

Sample	*D* (cm^2^ s^–1^) × 10^–8^	*µ* (cm^2^ V^–1^ s) × 10^–7^	*n* (cm^–3^) × 10^19^
CSPCFSN1	1.12	4.37	0.320
CSPCFSN2	1.17	4.55	5.39
CSPCFSN3	1.24	4.82	1.81
CSPCFSN4	1.71	6.64	48.2
CSPCFSN5	2.17	8.45	98.9

### Dielectric study

**Figure 5:**
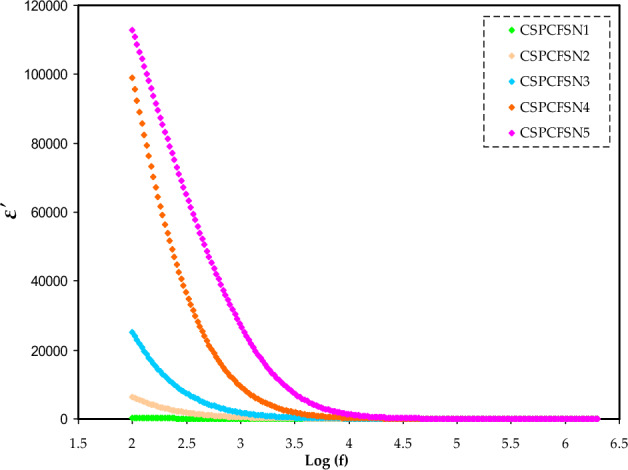
The plot of dielectric constant versus frequency.

**Figure 6:**
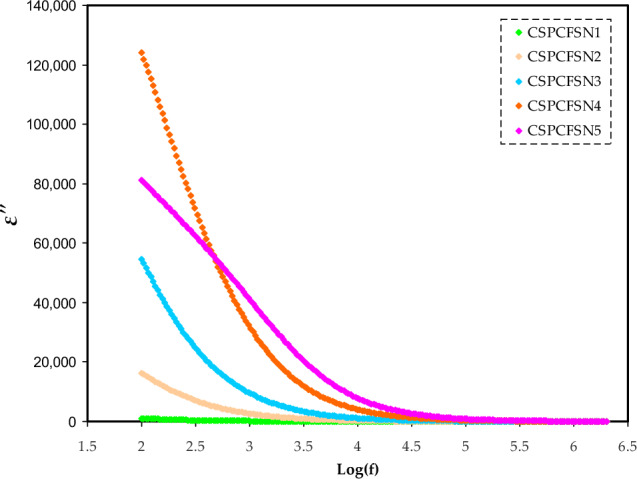
Trends of dielectric loss versus frequency.

To further verify the trend of conductivity with the loading of glycerol, the dielectric characteristics of PEs have been studied. Both the dielectric constant (ɛ′) and the dielectric loss (ɛ″) are measures of the energy dissipated by a material when ions pass through it. This study provides support for the notion that an increase in the quantity of free ions may lead to a higher conductivity value^40^. Both ɛ′ and ɛ″ are measured using the following equations:

9 $$ \varepsilon ^{\prime} = \left[ {\frac{Z^{\prime\prime}}{{\omega C_{^\circ } (Z^{{\prime}{2}} + Z^{{\prime\prime}{2}} )}}} \right], $$

10 $$ \varepsilon ^{\prime\prime} = \left[ {\frac{Z^{\prime}}{{\omega C_{^\circ } (Z^{{\prime}{2}} + Z^{{\prime\prime}{2}} )}}} \right], $$

where C_o_ is the capacitance in vacuum. Figures 5 and 6 show the ɛ′ and ɛ″ dependence on the loading glycerol content. CSPCFSN5 has the highest ɛ′ and ɛ″ values, particularly at low frequency region. The increased storage charges in the PEs indicate that the free ions number density has increased, thus improving the conductivity^41^. Aziz et al.^42^ reported that the ɛ′ values are in agreement with conductivity values. The high values of ɛ′ and ɛ″, at low frequencies are attributed to space charge and electrode polarization effects, which are characterized by non-Debye behavior^7^. On the other hand, as the electric field rapidly oscillates, resulting in the reduction of ion diffusion in the direction of the applied field, the values of ɛ′ and ɛ″ tend to decrease and stabilize at high frequencies. The decrease in polarization caused by charge accumulation is the primary reason for this drop, as noted in reference^43^.

The loss tangent (tan δ) is used to measure the relaxation behavior of the PEs. It is also referred to as the dissipation factor and represents the ratio of energy loss to energy stored in a periodic electric field^44^. The tan δ is calculated using the following equation:

11 $$ \tan \delta = \frac{{\varepsilon^{^{\prime\prime}} }}{{\varepsilon{\prime} }}. $$

**Figure 7:**
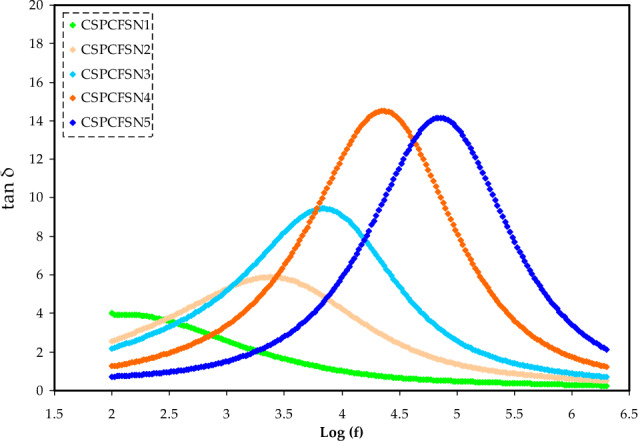
Tan δ plots for the samples considered in this work versus frequency.

The frequency dependence of tan δ on different PEs samples is depicted in Fig. 7. The tan δ at low frequency is observed to increase with increasing frequency as the reactive component (capacitive) is not as dominant as the active component (ohmic). However, at higher frequencies, the tan δ decreases due to the independence of the active component (ohmic) from frequency and the proportionate increase in the reactive component (capacitive) to frequency^45^. The tan δ maximum (tan δmax) shows the relaxation peak is shifted to the higher frequency for higher plasticized samples. The relaxation time (τ_r_) for the PEs is calculated using the following equation. Their values are presented in Table 5.

12 $$ \tau_{r} = \frac{1}{{\omega_{peak} }}, $$

where ω_peak_ is the relaxation peak angular frequency. The CSPCFSN5 has the smallest τ_r_ of 1.51 × 10^–5^ s, whereas the CSPCFSN1 has the largest τ_r_. The decrease in the τ_r_ is an indication that the polymer chains orient themselves with increasing amorphous phase upon loading glycerol^46^. Asnawi et al.^33^ results show a similar trend; samples with the highest conductivity showed the lowest values of τ_r_.

**Table 5:** Relaxation time (τ_r_) for the systems.

Sample	τ_r_ (s)
CSPCFSN1	6.76×10^–3^
CSPCFSN2	4.07×10^–4^
CSPCFSN3	1.51×10^–4^
CSPCFSN4	4.79×10^–5^
CSPCFSN5	1.51×10^–5^

### EDLC characteristics

#### Electrochemical stability measurement

**Figure 8:**
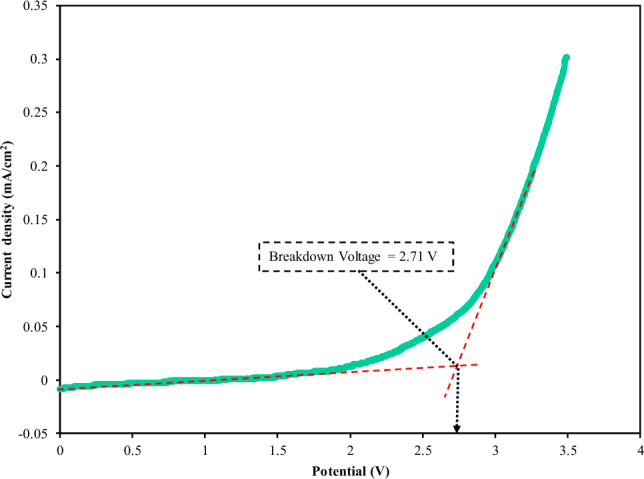
LSV for the CSPCFSN5 SPE film at 10 mV/s.

The electrochemical stability of any PE films intended to be used in energy storage devices such as EDLC needs to be examined using linear sweep voltammetry LSV^47^. Figure 8 shows the LSV of CSPCFSN5 at 10 mV/s where the breakdown voltage occurs at 2.71 V, which is the CSPCFSN5 decomposition. Brza et al.^28^ documented that the decomposition voltage of PVA/NH_4_SCN/glycerol electrolyte is 1.99 V, and the authors used the electrolyte for preparing EDLC. Our results show that the CSPCFSN5 is suitable for preparing an EDLC.

#### Cyclic voltammetry (CV) study

**Figure 9:**
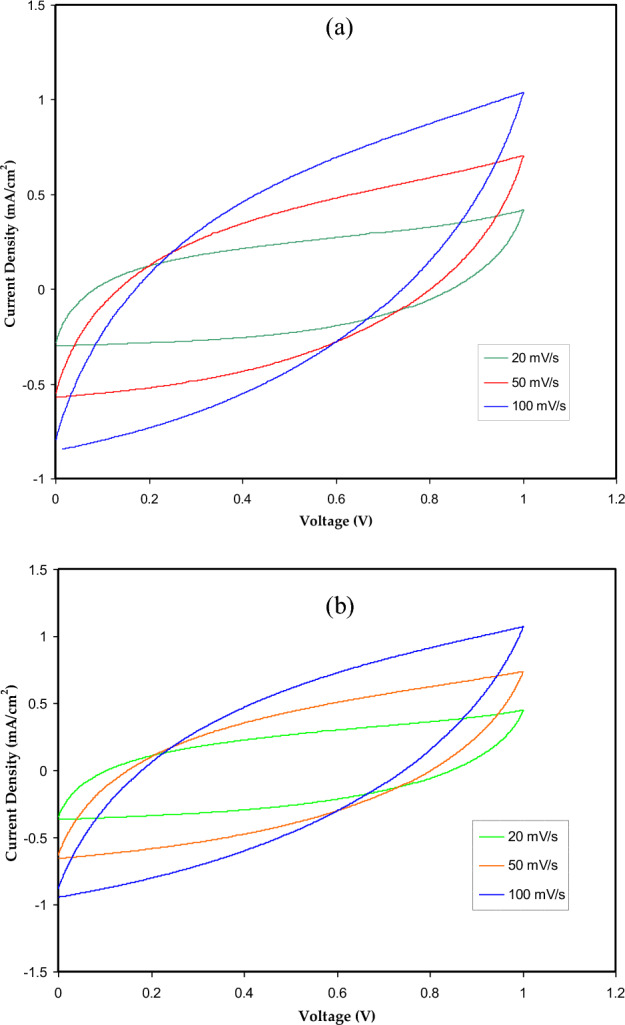
CV curve for the (**a**) CSPCFSN4 and (**b**) CSPCFSN5.

CV is a helpful method for elucidating the composition of the EDLC's anodic and cathodic interface charges^47,48^. Therefore, CV measurements were taken for CSPCFSN4 and CSPCFSN5 samples from 0 V to 1.0 V to evaluate their EDLC's performance. The results, as shown in Fig. 9a,b, reveal an approximately rectangular shape at a scan rate of 20 mV/s. This rectangular shape is an indication of constant ion diffusion within the EDLC, with minimal impact from ohmic resistance^49,50^. We used Eq. (11) to determine to determine the specific capacitance (Cs), at three different scan speeds, of the EDLC, and the results are shown in Table 6. According to the findings, the Cs falls as the scan rate rises because there is a greater energy loss and less charge storage on the electrodes^51^. Also, at low scan rates, the C_s_ is increasing because ions fill all the electrode’s vacant sites as they have sufficient time to diffuse through the vacant sites^52^. These results also highlight the importance of finding the optimal scan rate to balance energy loss and charge storage in EDLCs. At 50 and 100 mV s^−1^, the CV changes to a leaf-like shape as the current is postponed in getting a constant value owing to the equivalent series resistance (ESR) impact^28^. In literature, it has been reported that the ESR of the EDLC increases with more charge-discharge cycles^53,54^. However, when the scan rate is decreased, the impact of the ESR is reduced, leading to a roughly rectangular CV shape as the ESR contribution decreases^55,56^. This information can be useful for researchers in the development and optimization of EDLCs for various applications.

**Table 6:** Calculated Cs values for CSPCFSN4 and CSPCFSN5 samples.

Scan rate (mV/s)	Cs (F/g), CSPCFSN4	Cs (F/g), CSPCFSN5
100	3.23	3.31
50	4.93	5.26
20	7.84	8.63

**Figure 10:**
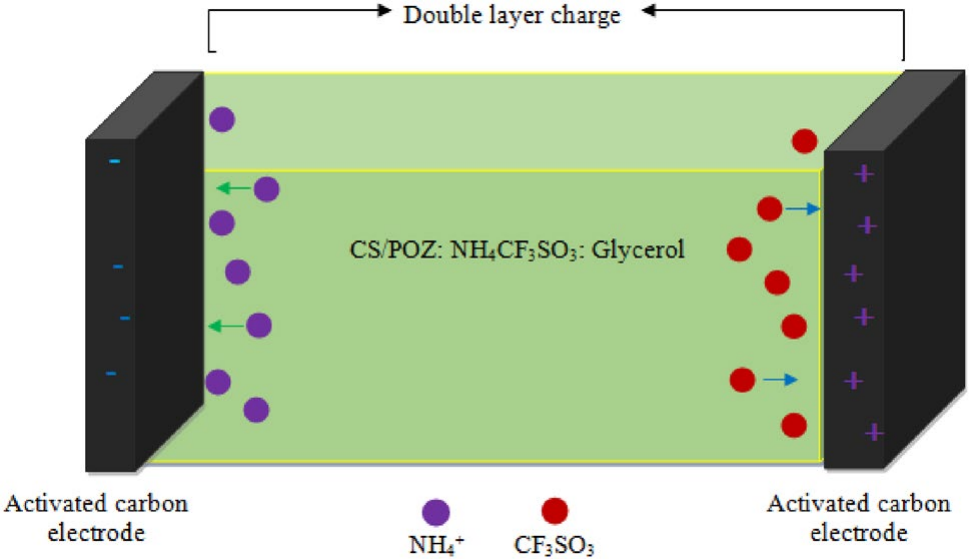
Diagram ion storage mechanism of CS:POZ:NH_4_CF_3_SO_3_:Gly based EDLC.

As seen in Fig. 9a,b and Table 6, CSPCFSN5 sample has higher C_s_ at the three scan rates and it is more resembling a rectangular shape in comparison to the CSPCFSN4 electrolyte system. This is because 50 wt.% of glycerol dissociates more salts into free ions in the CSPCFSN5 system, which leads to increased adsorption of more ions at the electrode and electrolyte interfaces. Figure 10 shows the electrochemical behavior of an EDLC based on CS:POZ:NH_4_CF_3_SO_3_:Gly, where NH^+^ and CF3SO3^–^ ions move in opposite ways toward the surface of the AC electrodes.

#### Galvanostatic charge-discharge (GCD) study

**Figure 11:**
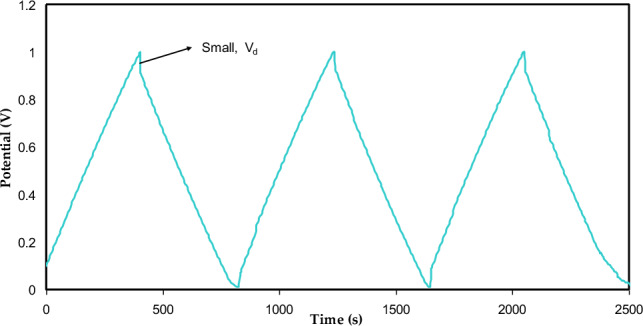
Charge-discharge curve for the CSPCFSN5 at current density 0.5 mA/cm^2^.

**Figure 12:**
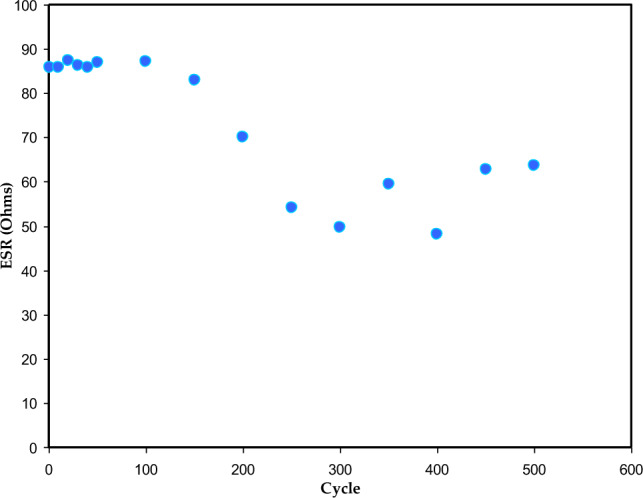
ESR versus cycle number for the CSPCFSN5.

The GCD at selected cycles of the EDLC for the CSPCFSN5 at 0.5 mA/cm^2^ is revealed in Fig. 11. The discharge parts are nearly linear, meaning that the EDLC has a capacitive behavior^57^. The drop voltage (V_d_) in the discharge curves of an EDLC is a crucial parameter that reflects the internal resistance of the system. The ESR of an EDLC is a measure of the total resistance within the device, which includes the resistance of the current collector, the resistance of the electrolyte, and the inter-fluid resistance between the electrolytes and current collectors^28,58^. Considerable weight is given to an EDLC's ESR in establishing its overall performance. As the voltage of an EDLC drops significantly during discharge due to a high ESR, its ability to store energy is compromised. While a high ESR causes a significant voltage drop, a low ESR improves the EDLC’s efficiency. By minimizing the ESR, the ionic transport can occur with minimal resistance, resulting in improved performance and higher energy storage capacity for the EDLC^52^. ESR is described by,

13 $$ ESR = \frac{{V_{d} }}{{i_{{}} }}, $$

where* i* is the applied current. The low values of V_d_ used in this work indicate that less energy is wasted in unnecessary heat production during the charging and discharging processes^59,60^. Figure 12 shows the ESR for 500 cycles, ranging from 87 to 50 Ω. This result is comparable to those reported in references^36,50^.

**Figure 13:**
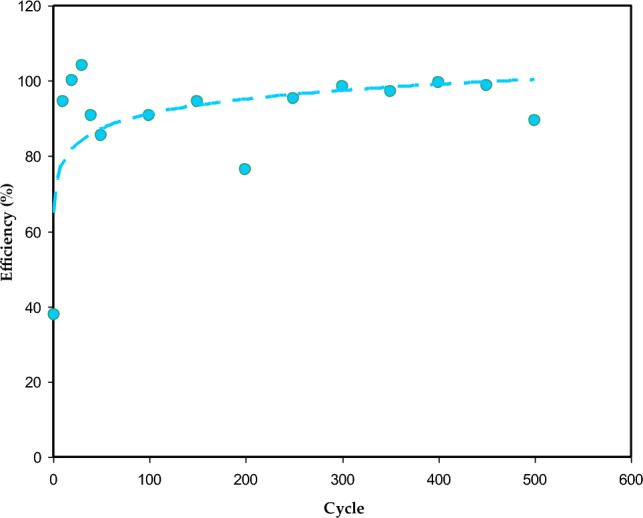
Coulombic efficiency η versus cycle number of CSPCFSN5.

As documented in Ref.^61^, EDLC efficiency is associated with its internal resistance. To examine the EDLC cycling stability, the Coulombic efficiency (η) values are calculated using the following equation:

14 $$ \eta = \frac{{t_{d} }}{{t_{c} }} \times 100\% , $$

where t_c_ and t_d_ are the charging time and discharging time, respectively. Figure 13 shows these results where the EDLC has η value of 88.2% during the 1st charging-discharging cycle. After several cycles, the η value increased and stayed almost unchanged at 90–100%, up to 500 cycles. Some scatter points can be observed above 100%, which may be ascribed to the fast charge-discharge process, but the efficiency is almost stable at 90–100%. At initial cycles, charge transport will be high, and their storage inside the porous area of activated carbon electrodes may be responsible of the higher value of efficiency. It is reported that the efficiency of nearly 90% shows that there is intimate contact between electrode and electrolyte^50^. This research shows that the EDLC using the CSPCFSN5 sample has superior cycling stability within 500 cycles.

**Figure 14:**
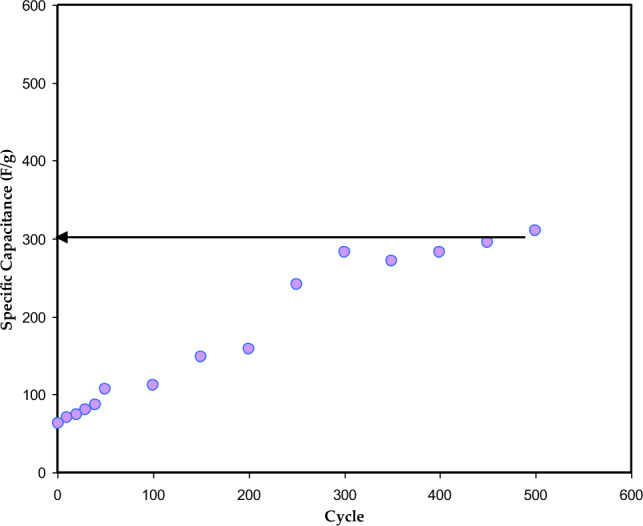
Cs versus cycle number for the CSPCFSN5.

The specific capacitance C_s_ of the EDLC, for each cycle is calculated using Eq. (15), and results are shown in Fig. 14. The C_s_ is ~ 70 F/g for the first cycle, and it is increased with increasing cycle number. The maximum C_s_ was found to be 300 F/g at 500 cycles, which is higher than those reported for liquid and gel electrolytes. The continuous increase of C_s_ value could be ascribed to the decrease of drop voltage (see Fig. 12). The decrease of ESR provides a better linear discharge curve and thus, more charge participate in the discharge process, which is essential for superior device performance. Consequently, the increase of C_s_ at high cycle numbers indicates that more ions are dissociating. In addition, over time and through repeated charge and discharge cycles, electrode materials can undergo a series of changes that enhance their performance. This transformative process leads to the creation of more available surface sites for ion adsorption, which can improve interactions between the electrode and electrolyte. Consequently, the effective surface area for ion storage and the overall capacitance can be enhanced. Additionally, the polymer electrolyte may experience polymer chain rearrangement or restructuring, resulting in better ion transport properties and increased capacitance. Furthermore, with continued cycling, electrolyte ions can redistribute and penetrate the structure of the electrode material, ultimately increasing the accessible ion-adsorption surface area. However, previous studies^50,62^ have shown a decrease in C_s_ as cycle numbers increase. This is because the ions tend to aggregate more during quick charge-discharge cycles, which blocks their motion and reduces their adsorption at electrolyte-electrode interfaces^39^. Hamsan et al.^63^ reported a C_s_ of 31 F/g at 0.2 mA/cm for EDLC using polyethylene PE electrolyte containing methylcellulose (MC), Potato starch (PS), NH_4_NO_3_, and glycerol^28^ determined a C_s_ of 18.3 F/g for EDLC using PVA/NH_4_SCN/glycerol electrolyte 450 cycles. C_s_ is given by the following formula:

15 $$ Cs = \frac{i}{xm}, $$

where *x* is the gradient of the discharge part.

**Figure 15:**
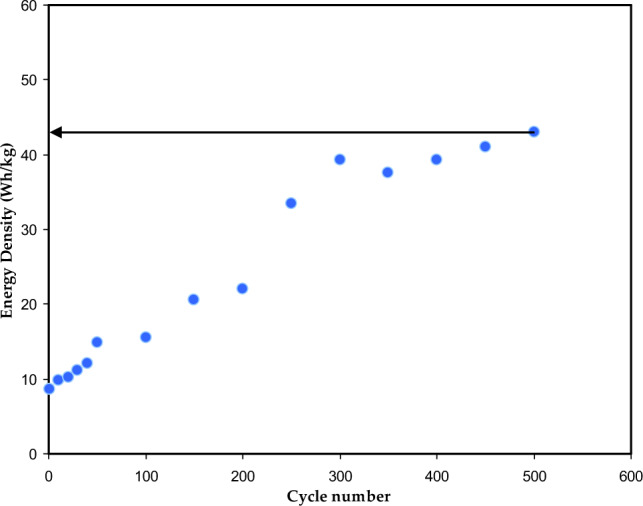
Energy density (*E)* versus cycle number for the CSPCFSN5.

**Figure 16:**
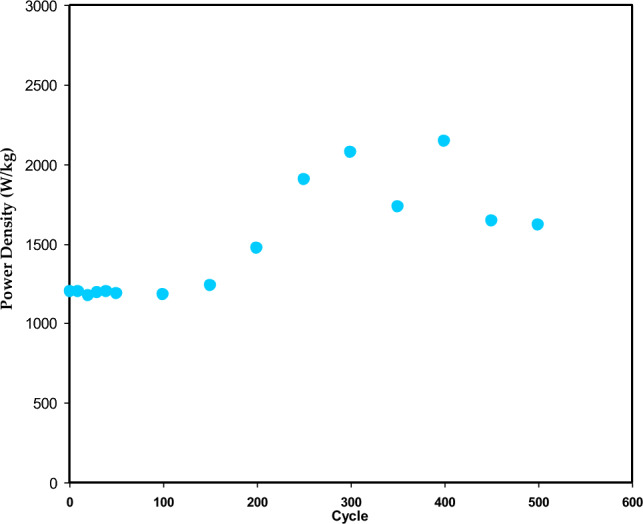
Power density (*P)* versus cycle number for the CSPCFSN5.

**Figure 17:**
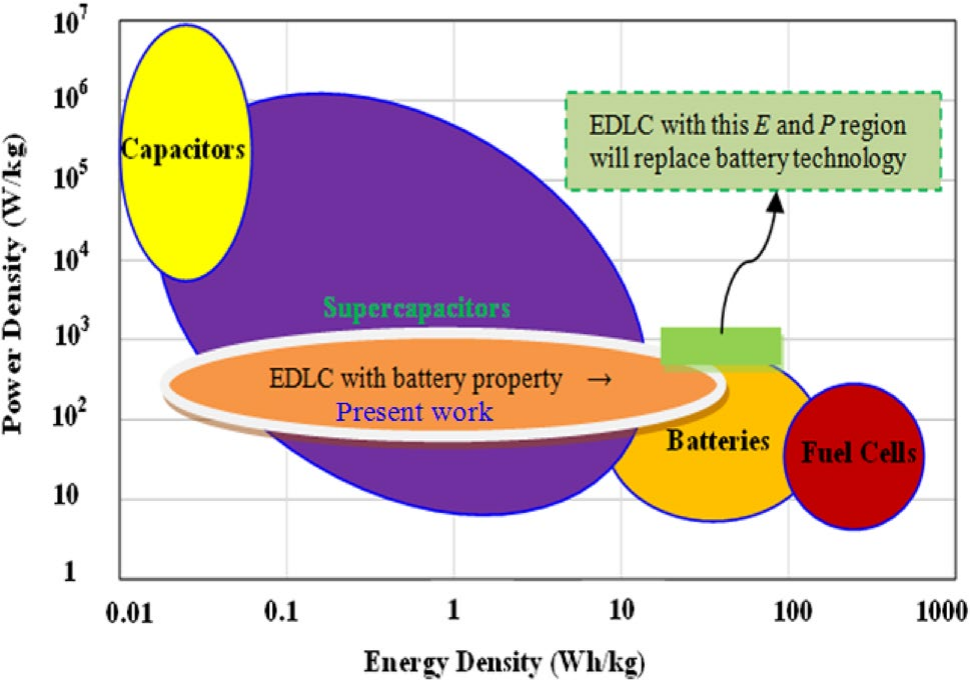
Ragone plot for various electrochemical devices.

Figures 15 and 16 show the energy density (E) and power density (P) of the EDLC, respectively. These are calculated using the following equations^39^:

16 $$ E = \frac{{C_{s} V^{2} }}{2}, $$

17 $$ P = \frac{{V^{2} }}{4m(ESR)}, $$

where V is the applied voltage. Upon careful observation, it is apparent that the *E* values illustrated in Fig. 15 demonstrate a similar pattern to that of the C_s_ values depicted in the previous figure. Specifically, the *E* values exhibit an increase from approximately 9.3 Wh/kg during the first cycle to 43 Wh/kg after 500 cycles. Hamsan et al.^63^ reported that the initial E value is 3.1 Wh/kg, which then stabilizes at approximately 2.2 to 2.3 Wh/kg after 1000 cycles. The authors attribute the decrease in E to an increase in internal resistance, resulting in energy loss during the charging-discharging mechanism. This suggests that fewer ions are aggregated through rapid charge-discharge cycles in the present study. To the best of our knowledge, the present study presents a novel discovery in the realm of EDLCs by documenting an *E* value that has not been previously reported in the literature. Table 7 presents the findings from studies on biodegradable polymer-based electrolytes used in EDLC devices. This outcome emphasizes that the *E* value depicted in the Ragone plot is not a constant, fixed quantity but rather is contingent upon the materials utilized in the system. The study emphasizes the importance of selecting biopolymers derived from non-toxic sources, green plasticizers, and a good proton-conducting salt to create clean energy storage solutions. Compared to Li-batteries, EDLCs have the advantage of being powered by smaller and safer protons, making it possible to replace Li-batteries with EDLCs if their lifetime can be controlled according to industry standards. Additionally, EDLCs have higher power densities than Li-batteries, even as represented in the Ragone plot, as shown in Fig. 17.

**Table 7:** Comparison of EDLC performance with activated carbon electrodes using different biodegradable polymer based electrolytes.

System	E (Wh/kg)	P (W/Kg)	*C*_*s*_ (F/g)	Cycles	Refs.
PVA+CH_3_COONH_4_+BmImBr	2.18	34660	21.89	500	^64^
PVA+dextran+NH_4_I	0.44	63	4.2	100	^65^
CS+MC+NH_4_I	1.1	578.55	9.97	100	^66^
CS+Dextran+NH_4_I	7.59	520.8	67.5	100	^67^
MC+NH_4_NO_3_+PEG	3.9	140	38	4	^36^
PVA+CH_3_COONH_4_+BmImCl	2.39	19.79	31.28	500	^54^
CS/MC+NH_4_F	9.3	1282	64.1	100	^68^
CS/PS+NH_4_F	0.57	155	6.85	100	^69^
Corn starch+LiClO_4_+SiO_2_	0.9	135	9.83	500	^70^
Corn starch+LiPF_6_+BmImPF_6_	2.53	7790	37.07	500	^71^
PVA+CH_3_COONH_4_+BmImTF	0.17	18370	3.35	500	^72^
CS:POZ: NH_4_CF_3_SO_3_:Gly	43	1800	300	500	This work

However, the main challenge facing EDLCs is their lower energy density compared to batteries. The results of the current study indicate that it is possible to achieve battery-like E values for EDLCs, which would make them more attractive to the energy storage industry. This would require redesigning the Ragone plot and conducting further research to optimize the performance of EDLCs based on biopolymers. To achieve a well-performing EDLC, careful preparation of the film in a dry state, proper encapsulation to ensure electrode-electrolyte contact and a thorough analysis of the results are necessary. The study's conclusion is that EDLCs composed of biopolymers represent a promising avenue for energy storage. It is recommended that further research be conducted in this domain to optimize the relevant parameters.

In 1st cycle, the P was nearly 1200 W/kg and increased to 2200 W/kg up to 400 cycles, as seen in Fig. 13. Previous studies have shown a significant decrease in P as the number of cycle’s increases^49,62^. This is due to the agglomeration of ions during fast charge-discharge cycles. It is crucial to observe that both E and P increased with increasing cycle number.

## Conclusion

In conclusion, it is reasonable to generate EDLC devices using plasticized biopolymers and a non-toxic salt [Chitosan:POZ:NH_4_CF_3_SO_3_:glycerol] with *E* and *P* values close to those in batteries. The addition of 50 wt.% glycerol (CSPCFSN5) resulted in an enhanced conductivity of 1.34 × 10^−4^ S cm^−1^, which was further validated by the conductivity and dielectric analysis trends. Using the EIS method, the diffusion coefficient, mobility, and number density of ions were measured to be 2.17 × 10^–8^ cm^2^ s^–1^, 8.45 × 10^–7^ cm^2^ V^–1^ s, and 9.9 × 10^20^ cm^–3^, respectively. The incorporation of a plasticizer reduced the relaxation time for proton conduction, as demonstrated by the asymmetrical tanδ plot and suppressed impedance semicircle, which indicated non-Debye ion transport behavior. The τ_r_ value was determined using the tanδ plot, and the CSPCFSN5 sample exhibited the lowest τ_r_ of 1.51 × 10^–5^ s. The film’s decomposition voltage was 2.71 V, demonstrating its suitability for use in EDLC devices. The CV plot showed a rectangular shape, indicating the capacitive behavior of the EDLC. The EDLC’s characteristics were analyzed through GCD measurements, and the device exhibited an energy density of 43 Wh/kg, a specific capacitance of 300 F/g, and a power density of 1800 W/kg after 500 cycles.

## Data Availability

The datasets used and/or analysed during the current study available from the corresponding author on reasonable request.
